# Have you really seen this illusory face before? Illusory faces bias recognition memory reports

**DOI:** 10.1098/rsos.261253

**Published:** 2026-10-21

**Authors:** Olga Kreichman, Limor Brook, Susan G. Wardle, Sharon Gilaie-Dotan

**Affiliations:** ^1^ School of Optometry and Vision Science, Bar-Ilan University, Ramat Gan, Israel; ^2^ The Gonda Multidisciplinary Brain Research Centre, Bar-Ilan University, Ramat Gan, Israel; ^3^ Laboratory of Brain and Cognition, National Institute of Mental Health, NIH, Bethesda, MD, USA; ^4^ University at Albany, State University of New York, Albany, NY, USA; ^5^ UCL Institute of Cognitive Neuroscience, London, UK

**Keywords:** image memory, face pareidolia, illusory faces, visual illusions, false memory, familiarity, signal detection theory, decision criterion

## Abstract

Visual memory is known to be influenced by objective image properties, including image size and contrast, as well as visual category. However, the influence of illusory perceptual experience on memory remains less understood. Here we investigated whether face pareidolia, a robust illusory phenomenon where certain stimuli elicit a face-like percept despite not being real faces, influences memory. While we assumed illusory faces would be remembered better than matched control images since they are surprising, interesting, draw more attention and may be associated with longer observation times, old-new recognition memory sensitivity was not consistently influenced by the face illusion. Surprisingly, decision criterion was more liberal for illusory faces than for other categories, and this effect scaled with illusory strength such that participants were more likely to report having seen illusory faces for stronger illusory face percepts. In a two-alternative-forced-choice recognition memory paradigm that minimizes criterion influence, we found that the tendency to report having seen illusory faces is present even for unseen images. Further analyses, involving confidence ratings indicated our findings probably reflect a heightened sense of familiarity for illusory faces and not genuine false memory. Our results suggest that during retrieval, illusory faces influence decisions independently of the actual memory trace.

## 1. Introduction

Objective aspects of images have been shown to influence visual memory, and this includes physical image properties (such as image size and contrast [1–3]) and the visual category the image is from [1–6]. These influences on visual memory may be driven directly by the psychological and neural resources devoted to encoding these image properties. So, for example, the physical image properties of retinal image size and contrast monotonically influence early stages of visual processing [7–15] such that higher contrast and bigger images lead to greater overall activity in the visual system (from the retina to retinotopic cortex [7–15]). Owing to this monotonic relation between the physical aspects of the image and the resources the visual system devotes to processing it, we have recently assumed and found that bigger and also higher-contrast images are better remembered [1–3,16].

As opposed to these objective, real-world properties of images and their influences on memory, less is known about the influences of illusory perceptual experiences on memory. Face pareidolia (FP) is a prevalent illusory phenomenon where certain stimuli appear to observers as face-like despite not being real faces (e.g. perceiving a car front as a face [17–20]). This phenomenon is robust and consistent across the population and has been studied extensively (e.g. [21–25]). Similar to additional illusory phenomena (e.g. [26–28]), FP also varies in its magnitude [18]. Since illusory faces are unusual, surprising, and may draw more attention and result in longer observation times, here we assumed that they would be remembered better than similar images that do not elicit an illusory face percept. We also assumed that this effect on memory would scale with the strength of the face illusion. In addition, it has recently been shown that beyond the typical higher-order visual cortex activity evoked when viewing non-face visual stimuli (e.g. lateral occipital complex (LOC) for objects and ‘parahippocampal place area’ (PPA) for places [29–31]), illusory faces also elicit activation in face-related visual regions [17,32]. We therefore also assumed that this recruitment of both face-related and non-face-related regions in high-order visual cortex may also contribute to better memory.

To test these predictions, we ran a series of old-new and two-alternative-forced-choice (2AFC) recognition memory experiments employing a naturalistic encoding task with a total of 281 participants. We measured memory for illusory face images with varying levels of the faceness illusion (with images not inducing illusory face percepts serving as content-matched control images for the illusory faces) and for faces and outdoor images. In the 2AFC experiments, we also included a critical target-absent condition with an unstudied illusory face appearing alongside an unstudied image from another category. This allowed us to examine whether there is a tendency to falsely report illusory faces more than chance, tapping into potential influences of illusory faces on false recognition.

Image memorability is considered an image-based trait that predicts the probability that people will remember an image ([33,34], but see [35]), and we assumed that it could be sensitive to the human perceptual experience of illusory faces. Recently, a machine learning model known as ResMem has been shown to successfully predict human memorability at the image level [36]. Since there have been many attempts to compare artificial neural networks with human visual cognition (e.g. [37]), here we examined whether model predictions for image memorability parallel human-based image memorability for different image categories. We estimated human image memorability from our behavioural data and compared it with the memorability predictions for the same images from the pre-trained residual neural network ResMem [36] that was trained on images from the LaMem [4] and the MemCat [38] datasets to predict image memorability [36,39]. This allowed us to test whether human memorability and the predictions of the neural network are sensitive to illusory faces. Notably, ResMem was not trained on illusory face images, so it was particularly interesting to compare its performance to human memorability for our image set.

## 2. Methods

### 2.1. Participants

In this study 281 participants aged 18–35 participated, with 95 in experiment 1 (aged 26.81 ± 4.09 years (M ± s.d.), 40 women), 117 in experiment 2 (aged 26.6 ± 4.74 years, 36 women), 49 in experiment 3 (aged 28.33 ± 4.56 years, 15 women) and 20 in experiment 4 (aged 28.3 ± 4.7 years, 9 women). Participants were recruited via the Prolific online platform (https://www.prolific.co/) with the following recruitment criteria: age (18–35 years), fluency in English, credible participation experience (at least 200 runs and an acceptance rate of 98% and above) and participation with a computer/laptop (not handheld devices). Each participant participated only once to ensure she/he was naive to the study's memory aspects. The results reported are based on 80 | 105 | 48 | 20 participants in experiment 1 | 2 | 3 | 4 that survived the performance-based exclusion criteria (see below). While recruitment criteria and participant demographics were available via Prolific, the identity of the Prolific participants was completely anonymized such that we were blind to any identifying details (each participant was known by her/his encrypted alphanumeric Prolific-created string). The experiments in this study were approved by the Bar-Ilan University Interdisciplinary Studies Unit ethics committee (approval number ISU202007003 granted on 14 August 2020) and by the Bar-Ilan University Institutional Review Board (approval number 060125410 granted on 6 January 2025). All the research was performed in accordance with the ethics committee and Declaration of Helsinki guidelines. Prior to their participation, participants were provided with an overall overview of the experiment (not exposing any information about the memory aspect of the experiment) as is common on Prolific. They were then asked to provide their informed consent to participate by agreeing that their data would be used (without identifying details) for research purposes before they could proceed to the actual experiment (upon their consent, they were automatically directed to the actual experiment hosted on Pavlovia (see below)). All participants reported having normal or corrected-to-normal vision before the experiment began. All participants were paid at the end of their participation according to the criteria set by Prolific (regardless of their level of performance in our study) and this was performed via the Prolific interface. To determine cohort size for these online experiments, given that data may be noisier than in laboratory-based experiments (see power analysis results in [1]), for experiments 1 and 2, we relied on a similar cohort size as in an earlier online memory study where we recruited approximately 60 participants for each version of each experiment [2] and full details of that study are available at https://osf.io/4ux9d/. For experiment 3, which had a different design, we initially recruited a cohort of 16 participants (‘cohort 1’), followed by a second (‘cohort 2’) and a third (‘cohort 3’) cohort of 16 and 17 participants, respectively, to test the replicability of the results. For experiment 4, we recruited 20 participants (‘cohort 4’) since the main goal was to only obtain and analyse confidence levels.

The participants' demographic information relied on information provided by Prolific (age, gender, country of origin and total number of approved experiments) and on self-reports collected in our experiments (vision status, handedness and highest level of education).

The experiments were created in PsychoPy (https://www.psychopy.org/online/, PsychoPy Builder v. 2021.2.3) and hosted on the online platform Pavlovia (https://pavlovia.org/).

### 2.2. Experiment 1

This experiment examined the effect of illusory faces on memory performance during naturalistic encoding [1–3]. Memory performance was estimated by a surprise old-new recognition memory test. Stimuli included four categories of images: faces, outdoor scenes, FP that induced a face illusion and content-matched control images (NoFP) that did not elicit a face illusion. There were two versions of this experiment.

#### 2.2.1. Stimuli

All images were real-world colour photographs. The face images consisted predominantly of a human face, typically with no visible body parts. Face images included individuals of varied age range (from babies to the elderly), gender and ethnic appearance, and portrayed a variety of expressions. Outdoor images did not include people in them and were from a variety of scenes (nature, urban, etc.). The face and outdoor images were from the ‘LaMem’ Dataset [4] and were the same as those used in earlier studies (experiment 5 in [1] and in [2]). The FP images were from multiple inanimate visual categories (mostly common objects, food and places) that induced an illusory face percept. The matched-control NoFP images were matched to the image content and visual appearance of each illusory face image as closely as possible but did not contain an illusory face. The NoFP images were manually matched in content to the FP images on a one-to-one basis and were all taken from experiment 2A of an earlier study [18]. The FP and NoFP images each had faceness ratings [18] from a scale of 0 (‘cannot see a face’) to 10 (‘easily see a face’) and these are available by version for the old-new (foil) images in table S1, in the supplementary material (see further image-specific details at https://osf.io/57u9w [40]).

A set of 480 images was used in this experiment, where each version consisted of only 320 of these (80 face and 80 outdoor images, which were the same across both versions, and 80 FP and 80 NoFP images, which were different across the two versions). The 160 FP images used in this study were chosen from those used in an earlier study [18] such that they were all with faceness ratings at or above six and were then randomly split into two sets of 80 FP images per version. For all the 80 FP images used in version1, their 80 content-matched control images appeared in version2. For all the 80 FP images used in version2, their 80 content-matched control images appeared in version1. An FP image and its unique matched control did not appear within the same version.

All images were resized to 900 × 900 pixels so that all images would occupy a consistent size on the participant's screen. It should be noted that only a subset of the original face and outdoor images did not have a 1 : 1 aspect ratio, which may have introduced some visible stretching for these images. Importantly, this did not affect the illusory face images or their matched controls, all of which had a 1 : 1 aspect ratio. All the experiment's uniformly sized images were then scaled to be displayed at 8.6° × 8.6° on the participant's screen (assuming a viewing distance of 60 cm; see more details below). Throughout the experiment, the screen background colour was black.

#### 2.2.2. Procedure

Participants were first presented with a demographics questionnaire (age range, gender, handedness, vision and highest level of education), and then proceeded to calibrate image size on their screen (using a screenscale module, see example at https://run.pavlovia.org/Wake/screenscale/) and to provide consent for how their data would be used (without identifying details) for research purposes. They then proceeded to the actual experiment. The experiment started with the exposure phase, where participants were asked to observe each image on the screen and to follow any instructions that may appear (without being informed about the memory aspect of the experiment, which was revealed later). The instructions were presented until the space key was pressed, after which the images were sequentially presented (2000 ms per image followed by a 500 ms inter-stimulus interval). This exposure phase included 160 images presented randomly (40 from each visual category: human faces, outdoors, illusory faces and matched control images; see figure 1). An unexpected attention check appeared after every 32 images (i.e. five times during the exposure phase), which consisted of a black screen with the instructions ‘Press SPACE as fast as you can’, and the experiment continued following the participant's response. These attention checks allowed us to evaluate the participants' engagement. After the exposure phase (160 presented images), additional instructions (for the test phase) appeared. Participants were presented with surprise old-new recognition memory instructions (to press the leftward arrow key if they recalled seeing the image earlier or the rightward arrow key if not) and were asked to press the shift key to proceed (the key needed to continue here was different from that used for the attention checks in order for the participants not to miss this instruction screen by quickly pressing the space key, assuming that it was another attention check). This was followed by a sequential presentation of 320 images for 500 ms each, in a random order (50% of the images were old; new images were matched for the categories of the old images). After the participant responded ‘old’ or ‘new’, the next image was presented (no feedback was provided). After every 100 images (i.e. three times during the test phase), a screen appeared that allowed participants to take a short break and to resume the experiment by pressing the space bar. The experiment ended with an ‘end of experiment’ screen, and participants were then directed back to Prolific with a completion code.

**Figure 1.**
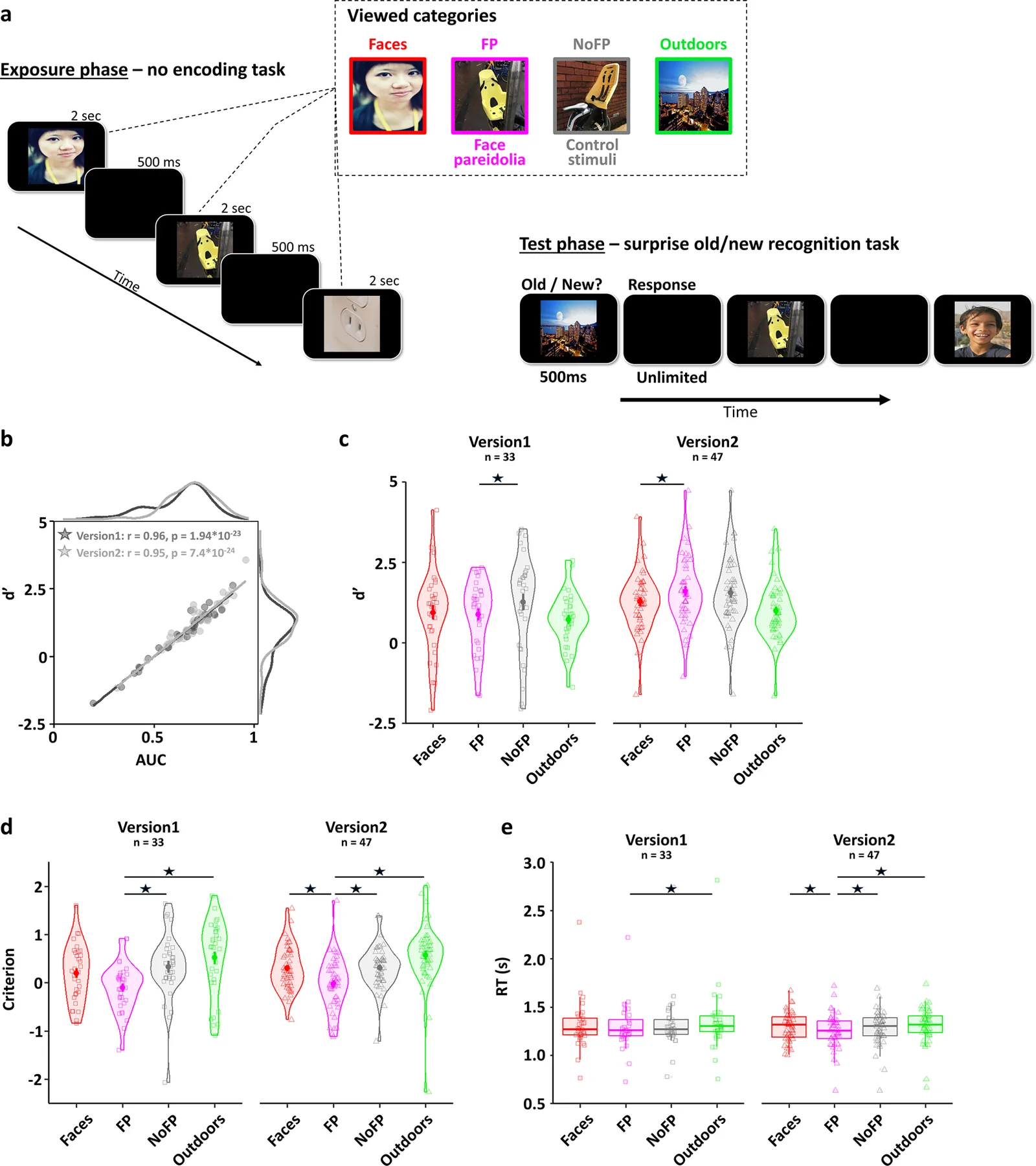
Old-new recognition memory decision criterion is more liberal for illusory face images than for other image categories: experiment 1 paradigm and results (*n*_(participants)_ = 80). (a) During naturalistic encoding (exposure phase), participants freely viewed 160 images while they were not informed of the memory aspect of the study. The 8.6° × 8.6° images were presented in random order (2 s per image) and were from four visual categories (40 images from each category: human faces (red), illusory faces (FP, magenta), matched control images (NoFP, grey) and outdoors (green); Methods). After the exposure phase, a surprise old-new recognition memory test phase followed with 320 randomly ordered images (160 old, 500 ms per image), and participants were asked to report for each image whether they recalled seeing it earlier or not. (b) Test-phase memory sensitivity (*d*') in the surprise old-new memory recognition task (y-axis) was significantly predictive of the area under the receiver operating characteristic (ROC) curve (AUC, x-axis). (c). Memory sensitivity (*d*') by condition (x-axis) and version (version1 on left, version2 on right) was not consistently affected by illusory faces (e.g. version1: *d*' of FP was lower than that of NoFP but not different from that of faces; version2: *d*' of FP was not different from that of NoFP and was higher than that of faces). Individual participant data annotated within each violin plot; the mean of each cohort is indicated by a filled circle and error bars (standard error of the mean (s.e.m.)). (d). Response criterion by condition (x-axis) and version (version1 on left, version2 on right) was significantly affected by illusory faces such that it was lower than that of all other categories in both versions (with marginal significance relative to that of faces in version1). Display notations as in (c). (e). Median reaction times (RTs) (in seconds) presented in boxplot notation by condition and version (as in c and d). Median is indicated by a line within each box plot. In version1 RTs for illusory faces were faster than those for outdoors; in version2 RTs for illusory faces were faster than those of all other categories. * denotes *p* < 0.05. See Results for more details.

### 2.3. Experiment 2

This experiment was designed very similarly to experiment 1 and was meant to replicate its results and test whether the effects found in experiment 1 are parametrically modulated by the level of the faceness illusion present in FP images. To that end, we used FP images with three varying levels of faceness ratings [18]: no illusory face percept (NoFP) that served as content-matched control images, low faceness (LowFP) and high faceness (HighFP) [18] as well as human face images (as used in experiment 1). See further details on https://osf.io/57u9w [40].

#### 2.3.1. Stimuli

All images were of the same type and content and taken from the same image sets as those used in experiment 1. The experiment included 320 images (the 80 human face images from experiment 1, 120 FP and 120 NoFP images). From the illusory face image set [18] we selected 60 FP images with the highest faceness ratings (HighFP, faceness ratings of 8.35 ± 0.05 (M ± s.e.m.)), 60 FP images with low faceness ratings (LowFP, faceness ratings of 4.98 ± 0.20) and the 120 content-matched control images for each of the FP images, which were confirmed by ratings as not containing an illusory face (60 control images for the HighFP (HighFP control), with faceness ratings of 0.22 ± 0.08 and 60 control images for the LowFP (LowFP control) with faceness ratings of 0.06 ± 0.03). Example images can be seen in figure 2.

**Figure 2.**
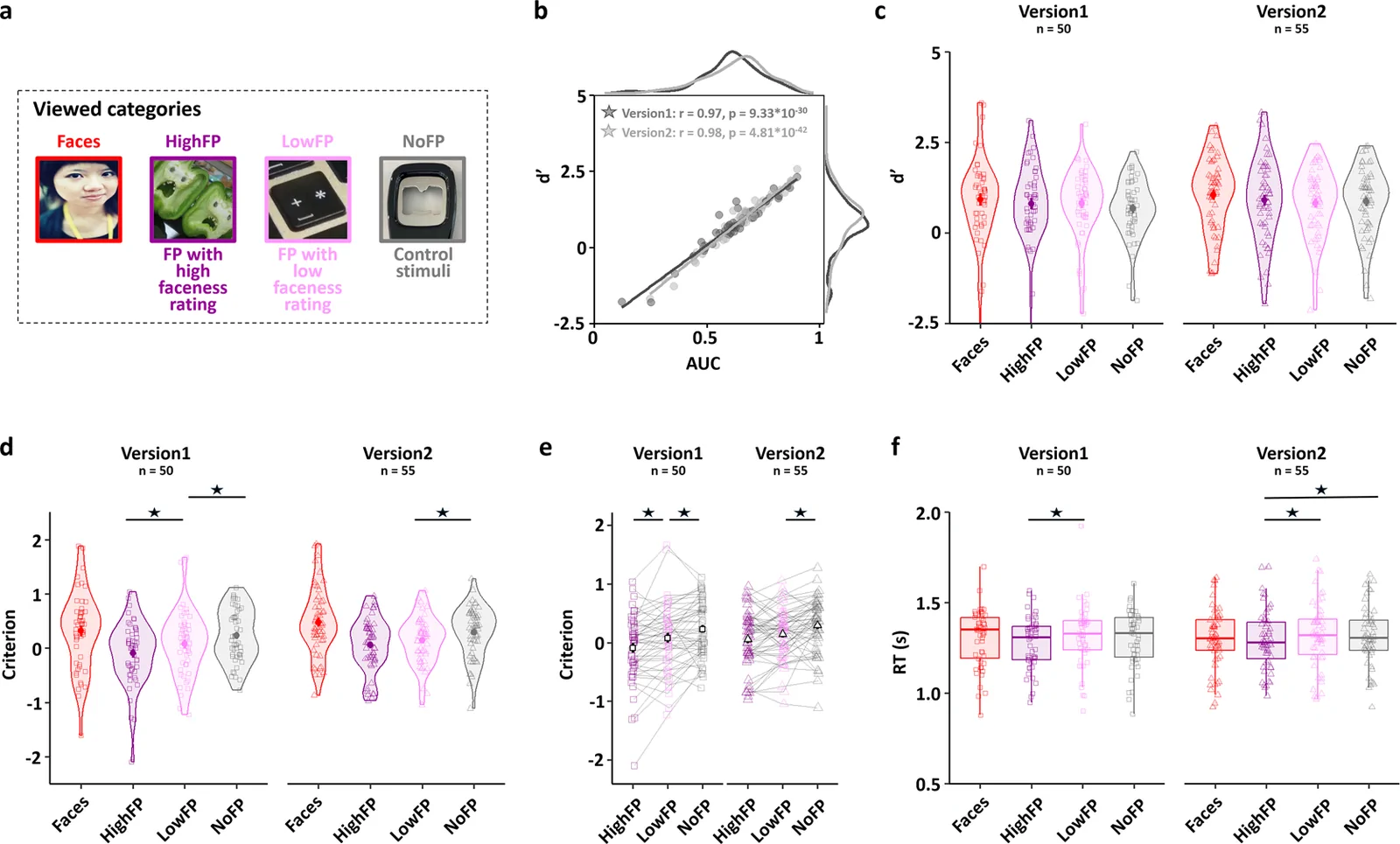
Old-new recognition memory decision criterion scales with illusory strength, becoming more liberal for stronger illusory face percepts: experiment 2 results (*n*_(participants)_ = 105) . (a) Example stimuli from the four visual categories of experiment 2 (Faces (red), high faceness illusory faces (HighFP, dark purple), low faceness illusory faces (LowFP, pink), matched control images (NoFP, grey); Methods). (b) Overall test phase memory sensitivity in the surprise old-new memory recognition task (*d*', on the y-axis) was significantly predictive of the area under the receiver operating characteristic (ROC) curve (AUC, x-axis). (c) Memory sensitivity (d') by condition (x-axis) and version (version1 on left, version2 on right) was not affected by illusory faces with no significant difference between NoFP, LowFP and HighFP. Individual participant data are annotated within each violin plot; the mean of each cohort is indicated by a filled circle and error bars (s.e.m.). (d) Response criterion by condition (x-axis) and version (version1 on left and version2 on right) was significantly affected by the level of illusory faces such that it was lower (more liberal) as the level of illusory faceness increased (criterion differences in version1: HighFP < LowFP < NoFP ≅ faces, and in version2: HighFP ≅ LowFP < NoFP < faces). Display conventions as in (c). (e) Individual criterion (y-axis) shifts according to illusory faceness level (x-axis) where individual data are connected by a grey line. Empty shapes denoted in black frame indicate the mean with error bars denoting s.e.m. (f) Median reaction times (RTs) (in seconds) by version and condition in boxplot notations. In both versions, RTs for HighFP were faster than those for LowFP (see Results for more details). * denotes *p* < 0.05. See Results for more details.

We created two versions of the experiment with comparable faceness ratings per experimental condition (HighFP, LowFP and NoFP control images). To that end, the 60 HighFP images were sorted in descending order of faceness ratings and were assigned to two sets by alternating rank of faceness ratings: odd-ranked images to Group A and even-ranked images to Group B. The same procedure was applied to the 60 LowFP images. Version1 included in the exposure phase 30 HighFP images of Group A, 30 LowFP images of Group A, 30 HighFP control images of Group B, 30 LowFP control images of Group B and 40 face images. All remaining images that were not presented in the exposure phase served as new (foil) images in the test phase. Similarly, version2 included in the exposure phase 30 HighFP images of Group B, 30 LowFP images of Group B, 30 HighFP control images of Group A, 30 LowFP control images of Group A and 40 face images (as those in version1). In the same manner, all remaining images that were not presented in the exposure phase served as new (foil) images in the test phase. Note that all 320 images appeared in each of the versions, but in each they served as either new or old. This means that an FP image did not appear with its content-matched control in the exposure phase, but during the test phase all FP images and their content-matched controls appeared in random order. Outdoor images were not included in this experiment since we aimed to keep the number of images (in the exposure phase, in the test phase and overall) as in experiment 1.

#### 2.3.2. Procedure

The procedure was as described above for experiment 1, apart from the following details. Participants were selected according to the criteria described above and we ensured that participants from experiment 1 did not participate by using a screening procedure available on Prolific. The exposure phase included 160 images presented in random order (40 faces, 30 HighFP, 30 LowFP, 30 HighFP and 30 LowFP control images) and the test phase included 320 images as described above.

### 2.4. Experiment 3

This experiment was designed to address three goals: (i) to measure memory performance in a fashion that is less influenced by decision (criterion) biases, (ii) to estimate false recognition memory for illusory faces, and (iii) to overcome potential image-specific results. To achieve the first goal, we employed a 2AFC task in the test phase. This 2AFC paradigm precludes the adoption of a liberal or conservative response criterion, since participants are required to choose between two alternatives on every trial [41,42]. To achieve the second goal, we included in the test phase, in addition to the valid trials (each presenting an old image and a new image), also critical target-absent trials where two new images were presented (one FP and one from another category). This allowed us to examine whether FP images result in higher false memory relative to false memory for other categories. To achieve the third goal, we applied a randomized image selection in each run, thereby overcoming potential image-specific effects.

#### 2.4.1. Stimuli

A set of 473 images was used in this experiment, where each run consisted of only 360 of these images. The whole image set included the 80 human face images used in experiment 1, the 80 outdoor images used in experiment 1, 140 illusory face images (FP) with faceness ratings greater than or equal to 7, and 173 matched control images with faceness ratings of 0 (NoFP); see further details about FP and NoFP images in [18]. From this image set, for each participant, a subset of 160 images was randomly selected to serve as the ‘old’ images (40 images randomly selected from each of the four categories), and additional, different 200 images were randomly selected to serve as ‘new’ images (20 from each of the four categories for the valid condition, and for the critical condition, 60 FP images and 20 from each of the other three categories). Example images can be seen in figure 3.

**Figure 3.**
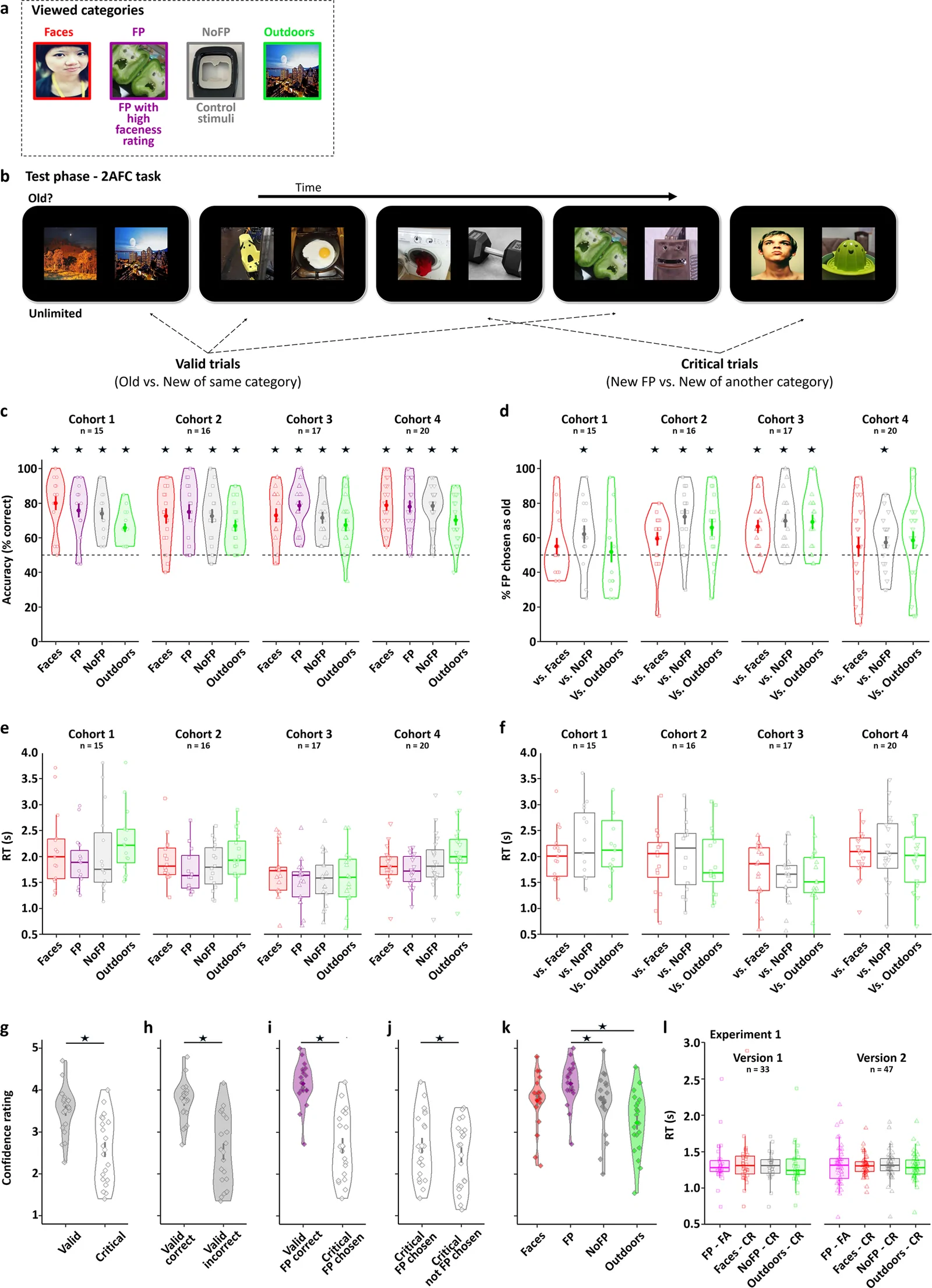
In the critical target-absent condition of a 2AFC recognition memory task, unseen illusory faces are falsely reported as seen at above-chance rates, but with lower confidence than for truly remembered illusory faces: experiments 3 (*n*_(participants)_ = 48) and 4 (*n*_(participants)_ = 20) paradigm and results. (a) Example stimuli from the four visual categories viewed (faces (red), illusory faces (FP, dark purple), matched control images (NoFP, grey) and outdoors (green); Methods). (b) Test phase in the 2AFC paradigm included valid trials (seen (target) and unseen (foil) image pair from the same category presented side by side) and critical trials (two unseen images, one FP and one from another category presented side by side; Methods). (c) Test-phase accuracy for valid trials by category and by experimental cohort (cohorts 1–3: experiment 3, cohort 4: experiment 4) revealed recognition memory was significantly above chance for all categories. Display conventions as in figure 1c. (d) Proportion of falsely reported unseen FP images (relative to unseen images from other categories). In all cohorts, FPs were reported as seen more than the control (NoFP) images. (e) Median reaction times (RTs) for correct responses on valid trials. (f) Median RTs for FP choices on critical trials. (g–k) Experiment/cohort 4 confidence ratings (1 (‘very unsure’) to 5 (‘very sure’); n=20). (g) Confidence of valid trials was higher than that of critical trials and (h) confidence of valid correct trials was higher than that of valid incorrect trials. (i) Confidence for remembered FP images (valid FP correct trials) was higher than that of falsely remembered FP images (FP chosen on critical trials), and at the same time (j) confidence for falsely remembered FP images (FP chosen on critical trials) was higher than that of falsely remembered images from other categories (other category chosen on critical trials). (k) On valid trials, confidence for FP correctly remembered images was higher than that of correctly remembered images from other categories (see Results). (l) Based on data from experiment 1, RTs for falsely recognized FP images were not consistently different from those of correctly rejected images from other categories. This suggests the above-chance FP reports in the critical trials probably reflected genuine false familiarity (see Results). * denotes *p* < 0.05.

#### 2.4.2. Procedure

Participants were recruited according to the criteria described above, and we ensured that participants from experiment 1 or 2 did not participate in this experiment by using a screening procedure available on Prolific. The exposure phase followed the same procedures as in experiment 1 and included 160 images (40 from each visual category) presented in random order. After the exposure phase (the 160 presented images), additional instructions (for the test phase) appeared. Participants were presented with surprise 2AFC recognition memory instructions (to use their mouse in each trial to choose the image they recall seeing earlier from the pair of presented images). This was followed by a sequential presentation of 140 pairs of images, each presented until a response was given. There were 80 valid pairs (20 from each category) that included an already seen image (‘old’) and a novel image (‘new’) of the same category. There were also 60 critical pairs that each presented a new FP image besides a new image from another category (20 trials with a new outdoor image, 20 trials with a new face image and 20 trials with a new NoFP image). The position of the old image in the valid pairs and of the FP image in the critical pairs was random (on the right or left of the screen) to ensure that any spatial positional bias would not systematically influence our findings. The first 20 trials were always valid and the rest of the trials were mixed between the remaining 60 valid and 60 critical trials. No feedback was provided and after the first 100 trials (i.e. once during the test phase), a screen appeared that allowed participants to take a short break and resume the experiment by pressing the space bar. The experiment ended with an ‘end of experiment’ screen and participants were then directed back to Prolific with a completion code.

### 2.5. Experiment 4

This experiment was designed to further test the replication of experiment 3 results and to examine whether confidence levels for the memory reports for falsely recognized images in the critical trials were similar to those for correctly recognized images in the valid trials. Therefore, this experiment replicated experiment 3, and in addition, in the test phase, after each trial, we also obtained per-trial confidence ratings. The confidence ratings screen presented a Likert scale between 1 (‘very unsure’) and 5 (‘very sure’) and was presented until a confidence response was selected.

### 2.6. Analyses

Data from each participant were recorded as a comma-separated values (CSV) file, which included response times and accuracy for each presented image for experiments 1 and 2. For experiments 3 and 4, data also included the two presented images in each trial and the selected image. For experiment 4, the data also included the reported confidence level and its reaction time (RT). These CSV files were then analysed with R (RStudio 2021.9.0.351 [43]) in-house scripts to obtain per-condition results across participants, per-image (memorability-related) results as well as statistical analyses.

In experiments 1 and 2, memory performance was estimated for each participant by the accuracy for the old images per experimental condition (aka hits in signal detection theory), by the memory sensitivity (*d*'), decision criterion (response bias) and overall area under the RT-based receiver operating characteristic (ROC) curve [2,44]. RTs were analysed only for correct trials. In experiments 3 and 4, memory performance was estimated by the 2AFC accuracy for the valid trials and by the proportion of trials in which the FP was chosen as ‘old’ in the critical target-absent trials. RTs for valid trials were determined according to the correct responses only and for the critical trials from the trials where FP was chosen as ‘old’. In experiment 4, we also examined confidence ratings by condition type (valid or critical), by category and by correctness.

Repeated measures ANOVAs were performed using the R anova_test and get_anova_table functions, where get_anova_table automatically applies the Greenhouse–Geisser sphericity correction only to factors violating the sphericity assumption (i.e. Mauchly's test significant *p*-value ≤ 0.05). Effect size estimates were obtained using the anova_test function with the effect.size parameter set to ‘ges’, which provides generalized eta squared values. A post hoc Bonferroni/Dunn test was used (note that we report a *p*-value of 1 when R's post hoc multiple comparison correction produces a corrected value of *p* ≥ 1). For RT analyses, we used a Friedman test (friedman.test), and significant main effects were followed up with pairwise comparisons using the Wilcoxon signed-rank test (pairwise.wilcox.test, Bonferroni corrected). We also tested whether the proportion of FP choices in the critical condition was above chance (comparing performance with 50% chance) using the Wilcoxon signed-rank test.

Human memorability (based on experiment 3 data) was estimated for each image as the proportion of participants who chose it as old when it was old (*p*(Hits)) minus the proportion of participants who chose it as old when it was new (*p*(false alarms)) and it is thus presented in arbitrary units. This was possible only for experiment 3, where each condition's images (e.g. old or new) were randomly selected from each category's pool of images, thereby each image could be old for some participants and new for others. The memorability analysis included only images that appeared for at least 12 different participants such that for at least six participants they were presented as old images and for at least six other participants they were presented as new.

For comparison with human memorability, we calculated model predictions for the memorability of the individual images used in experiment 3 with the residual neural network ResMem [36]. We used the Python package ‘resmem’ (https://github.com/Brain-Bridge-Lab/resmem, https://pypi.org/project/resmem/) to access the pre-trained neural network model and wrote a custom wrapper script in Python to systematically calculate a predicted memorability score (on a scale from 0 to 1) for each of our images. These ResMem memorability results were then subjected to a statistical ANOVA by image category and a correlation analysis with human-based memorability scores on an image-by-image basis.

Performance-based exclusion criteria (independent of our research question) were applied to experiments 1 and 2 to reduce potentially noisy data. We first excluded participants with 0% performance for the old or new images and 0 or 100% in the new condition (no participants were excluded), then participants who failed our *attention checks* (i.e. failed one or more of the five attention checks (taking longer than 8 s to respond to the first attention check or more than 3 s to respond to any of the four consecutive attention checks)). This resulted in 15 participants being excluded from experiment 1 and 12 from experiment 2. In experiments 3 and 4, we excluded participants who consistently clicked the same side of the screen throughout the test phase and this resulted in the exclusion of one participant from experiment 3.

Overall, 15 participants were excluded from experiment 1 (seven from version1 and eight from version2) out of 95 that participated (15.79%), 12 participants from experiment 2 (nine from version1 and three from version2) out of 117 that participated (10.26%), one participant from experiment 3 (2.04%), and none from experiment 4 (0%). Therefore, the reported results of experiment 1 are based on the remaining 80 participants after exclusion, the reported results of experiment 2 are based on the remaining 105 participants after exclusion, the reported results of experiment 3 are based on 48 participants and the results of experiment 4 are based on 20 participants. Note that all details and data, including those of the excluded participants, are available on OSF at https://osf.io/57u9w [40].

## 3. Results

### 3.1. Experiment 1

To test our hypotheses that illusory face images are remembered better than non-face images with similar visual properties and perhaps more than real face images, we ran experiment 1, where participants freely viewed images of human faces, outdoor scenes (without people), illusory faces (FP) and control images for the FP images that were content-matched but did not induce an illusory face percept (NoFP; figure 1a).

We first examined whether overall memory was above chance (or guessing) behaviour (figure 1b). To that end, we compared the overall *d*' with the chance level (i.e. to 0) and also the area under the ROC curve (AUC) to the chance level (i.e. to 0.5). Both measures were significantly above chance (version1: overall *d*': 0.83 ± 0.17 (M ± s.e.m.), 95% CI [0.49, 1.18], *t*(32) = 4.92, *p* = 2.49 × 10^–5^; AUC: 0.64 ± 0.03, 95% CI [0.58, 0.70], *t*(32) = 5.02, *p* = 1.88 × 10^–5^; version2: overall *d*': 1.25 ± 0.11, 95% CI [1.02, 1.48], *t*(46) = 10.87, *p* = 2.66 × 10^–14^; AUC: 0.70 ± 0.02, 95% CI [0.67, 0.74], *t*(46) = 11.42, *p* = 5.03 × 10^–15^; one-sample *t*-test, 2-tailed). We also found that *d*' and AUC were significantly predictive of each other (version1: *r* = 0.98, *r*^2^ = 0.96, *t*(31) = 27.7, *p* = 1.94 × 10^–23^; version2: *r* = 0.95, *r*^2^ = 0.9, *t*(45) = 19.8, *p* = 7.4 × 10^–24^), similarly to our findings in earlier studies [2,16]. This indicates that during this naturalistic encoding experiment, memory sensitivity surpassed guessing behaviour.

To examine whether we could collapse the results across the two experimental versions, we ran a two-way ANOVA on memory sensitivity with experimental version and visual category as main factors. Since a significant interaction between version and category on memory sensitivity (*d*') was found (version: *F*(1,78) = 3.27, *p* = 0.07, *ŋ*^2^*_G_* = 0.034; category: *F*(2.59,201.71) = 18.09, *p* = 1.95 × 10^–9^, *ŋ*^2^*_G_* = 0.034; interaction: *F*(2.59,201.71) = 3.84, *p* = 0.014, *ŋ*^2^*_G_* = 0.008), we report the results of each version separately.

An effect of category on memory sensitivity (*d*') was found in both versions (version1: *F*(2.45,78.55) = 6.28, *p* = 0.002, *ŋ*^2^*_G_* = 0.028; version2: *F*(3,138) = 18.39, *p* = 4.26 × 10^–10^, *ŋ*^2^*_G_* = 0.06; figure 1c) but it did not reflect similar and consistent between-category memory sensitivity modulations across versions. In version1, FP memory sensitivity was not different from that of faces (FP versus faces: *p* = 1) but was lower than that of NoFP (FP versus NoFP: *p* = 0.014). In addition, outdoors memory sensitivity was also lower than that of NoFP (outdoors versus NoFP: *p* = 0.012). In version2, FP memory sensitivity was higher than that of faces (FP versus faces: *p* = 0.001), but not different from that of NoFP (FP versus NoFP: *p* = 1). In addition, outdoors memory sensitivity was again lower than that of NoFP (*p* = 4.9 × 10⁻⁵). In summary, FP memory sensitivity was not consistently higher than that of NoFP or of face images. In addition, outdoor images' average memory sensitivity was consistently the lowest, in line with earlier studies [2,16].

The results of the decision criterion were different and showed consistent effects for FP images (figure 1d). There was a significant effect of category on decision criterion in both versions (version1: *F*(2.04,65.19) = 10.38, *p* = 1.1 × 10⁻⁴, *ŋ*^2^*_G_* = 0.113; version2: *F*(2.35,107.95) = 21.3, *p* = 2.14 × 10^–9^, *ŋ*^2^*_G_* = 0.141) that reflected a consistently lowest decision criterion for FP images across the two versions. Specifically, in version1 FP decision criterion was significantly lower than that of most categories (versus faces: *p* = 0.1, versus NoFP: *p* = 0.006, versus outdoors: *p* = 0.003), and in version2 this was significant for all categories (versus faces: *p* = 1.3 × 10⁻⁴, versus NoFP: *p* = 5.8 × 10⁻⁶, versus outdoors: *p* = 7.7 × 10⁻⁷). Since the criterion for FP was lowest we also examined whether it was significantly positive and found that it was not (version1: –0.10 ± 0.08 (M ± s.e.m.), 95% CI [–0.27, 0.07], *t*(32) = –1.21*, p* = 0.23; version2: –0.03 ± 0.08, 95% CI [–0.19, 0.13], *t*(46) = –0.38*, p* = 0.71; one-sample *t*-test, two-tailed). This was in contrast to the positive criterion found for all other categories, with faces in version1 being marginally positive (version1: faces: 0.20 ± 0.10, 95% CI [–0.007, 0.40], *t*(32) = 1.97, *p* = 0.058; NoFP: 0.33 ± 0.12, 95% CI [0.08, 0.57], *t*(32) = 2.729, *p* = 0.01; outdoors: 0.52 ± 0.13, 95% CI [0.24, 0.79], *t*(32) = 3.85, *p* = 5 × 10⁻^4^; version2: faces: 0.30 ± 0.07, 95% CI [0.17, 0.44], *t*(46) = 4.59, *p* = 3.44 × 10^–5^; NoFP: 0.31 ± 0.06, 95% CI [0.18, 0.44], *t*(46) = 4.8, *p* = 1.50 × 10^–5^; outdoors: 0.57 ± 0.1, 95% CI [0.38, 0.76], *t*(46) = 5.97, *p* = 3.24 × 10^–7^; one-sample *t*-test, two-tailed). In summary, the decision criterion for FP images was significantly lower than that found for all other categories and this was consistent in both versions. Furthermore, the FP decision criterion did not differ significantly from zero, whereas the criterion for all other categories was significantly positive.

Additional analyses included RTs that were significantly affected by category (version1: $\chi^{2}$(3) = 8.11, *p* = 0.044; version2: $\chi^{2}$(3) = 30.2, *p* = 1.2 × 10⁻⁶; figure 1d) and this was driven by slower RTs for outdoor images (version1, versus FP: *p* = 0.024; version2, versus FP: *p* = 5.2 × 10⁻⁵, versus NoFP: *p* = 0.036 and versus faces: *p* = 0.044). Furthermore, accuracy analyses for old and for new images can be found in the supplementary material along with individual data at https://osf.io/57u9w [40].

### 3.2. Experiment 2

In experiment 1, we found that the decision criterion was significantly lower for FP images relative to their matched control images. However, the FP images in experiment 1 had high faceness ratings (greater than or equal to 6), whereas the matched control (NoFP) images had floor faceness ratings (close to 0). Therefore, here we aimed to examine whether the decision criterion was parametrically modulated by the magnitude of the induced face illusion. To that end, in this experiment, we used illusory faces of varying faceness ratings from high faceness to floor faceness levels (supplementary material, table S1) and human face images (figure 2a). The floor faceness images served as content-matched control images for the high and low faceness illusory face images. There were no apparent differences in content and no significant differences in faceness ratings between the matched control images of the HighFP and the matched control images of the LowFP conditions (version1: *t*(30.01) = –1.70, *p* = 0.1; version2: *t*(42.65) = –0.80, *p* = 0.43; non-repeated two-tailed *t*-test) and therefore these were treated in the analysis as one category (NoFP).

We first replicated the results of experiment 1, finding that overall memory was above chance/guessing behaviour (figure 2b). Both overall *d*' and AUC were significantly above chance level (version1: overall *d*': 0.73 ± 0.12 (M ± s.e.m.), 95% CI [0.50, 0.97], *d*' above 0: *t*(49) = 6.23, *p* = 1.02 × 10^–7^; overall AUC: 0.62 ± 0.02, 95% CI [0.58, 0.66], AUC above 0.5: *t*(49) = 5.73, *p* = 6.15 × 10^–7^; version2: overall *d*': 0.85 ± 0.12, 95% CI [0.61, 1.09], *d*' above 0: *t*(54) = 7.04, *p* = 3.55 × 10^–9^; overall AUC: 0.64 ± 0.02, 95% CI [0.60, 0.68], AUC above 0.5: *t*(54) = 7.28, *p* = 1.43 × 10^–9^; one-sample *t*-test, two-tailed). *d*’ and AUC were also found to be significantly predictive of each other (version1: *r* = 0.97, *r*^2^ = 0.93, *t*(48) = 25.8, *p* = 9.33 × 10^–30^; version2: *r* = 0.98, *r*^2^ = 0.97, *t*(53) = 41.4, *p* = 4.81 × 10^–42^). These results indicate that memory sensitivity in this experiment during a naturalistic encoding paradigm surpassed guessing behaviour, in line with the results of experiment 1.

We also examined here the effect of category on memory sensitivity to test whether it was parametrically affected by the level of the faceness illusion. A two-way ANOVA testing for the effects of both category and experimental version on memory sensitivity revealed a significant effect of category without an effect of version or interaction between version and category (category: *F*(3,309) = 5.32, *p* = 0.001, *ŋ*^2^*_G_* = 0.006; version: *F*(1,103) = 0.31, *p* = 0.58, *ŋ*^2^*_G_* = 0.003; interaction: *F*(3,309) = 0.91, *p* = 0.44, *ŋ*^2^*_G_* = 0.001). However, to be consistent with the presentation of experiment 1 results, we ran separate one-way ANOVA per version to test the effect of category on memory sensitivity (*d*') and found a marginal effect in version1 and a significant effect in version2 (version1: *F*(3,147) = 2.5, *p* = 0.062, *ŋ*^2^*_G_* = 0.007; version2: *F*(3,162) = 3.93, *p* = 0.01, *ŋ*^2^*_G_* = 0.008). Importantly, as in experiment 1, where no consistent difference in memory sensitivity was found between FP and NoFP, here too we did not find that memory sensitivity was affected by the level of the faceness illusion. However, we did find that the category effect on memory sensitivity reflected the highest *d*' for faces that was significantly above that of NoFP in both versions (version1: *p* = 0.046, version2: *p* = 0.041) and also higher than that of LowFP in version2 (*p* = 0.006).

Consistent with the results of experiment 1, here we found an effect of category on decision criterion in both versions (version1: *F*(2.27,111.04) = 14.28, *p* = 1.03 × 10^–6^, *ŋ*^2^*_G_* = 0.066; version2: *F*(2.27,122.42) = 17.73, *p* = 4.35 × 10^–8^, *ŋ*^2^*_G_* = 0.091) and this can be seen in figure 2d. In both versions memory criterion for HighFP was on average the lowest, followed by a higher criterion for LowFP, then NoFP and the highest criterion on average was found for faces. Specifically, in version1 criterion for HighFP was lower than that of LowFP (*p* = 0.007), criterion for LowFP was lower than that of NoFP (*p* = 0.028), and criterion for NoFP was not significantly different from that of faces (*p* = 1). In version2 criterion of HighFP was similar to that of LowFP (*p* = 0.39), criterion for LowFP was lower than that for NoFP (*p* = 0.016), and the criterion for NoFP was lower than that for faces (*p* = 0.021). Since the criterion for HighFP was the lowest, here too we examined whether it was significantly positive and found that it was not positive when the faceness illusion was strongest (HighFP: version1: –0.093 ± 0.08 (M ± s.e.m.), 95% CI [–0.259, 0.072], *t*(49) = –1.14, *p* = 0.26; version2: 0.06 ± 0.07, 95% CI [–0.07, 0.19], *t*(54) = 0.87, *p* = 0.39), when the faceness illusion was moderate it was inconsistently positive (i.e. not-significantly positive in version1 and significantly positive in version2; LowFP: version1: 0.08 ± 0.08, 95% CI [–0.09, 0.24], *t*(49) = 0.96, *p* = 0.34; version2: 0.14 ± 0.06, 95% CI [0.03, 0.26], *t*(54) = 2.47, *p* = 0.02; one-sample two-sided *t*-test), and it was significantly positive when there was no faceness illusion (NoFP: version1: 0.23 ± 0.07, 95% CI [0.09, 0.37], *t*(49) = 3.36, *p* = 0.0015; version2: 0.29 ± 0.06, 95% CI [0.17, 0.42], *t*(54) = 4.67, *p* = 2 × 10^–5^; Faces: version1: 0.32 ± 0.1, 95% CI [0.11, 0.52], *t*(49) = 3.16, *p* = 0.0027; version2: 0.47 ± 0.08, 95% CI [0.31, 0.64], *t*(54) = 5.73, *p* = 4.5 × 10^–7^; one-sample two-sided *t*-test). Overall, the decision criterion was parametrically reduced with increasing levels of faceness in illusory faces (figure 2e), indicating that participants were more likely to falsely report remembering stronger examples of FP than weaker examples. In addition, as in experiment 1, decision criterion for high-faceness illusory faces was not positive, while it became positive as the level of the faceness illusion weakened.

Additional analyses included RTs, which were significantly affected by category (version1: $\chi^{2}$(3) = 11.9, *p* = 0.0077; version2: $\chi^{2}$(3) = 14.2, *p* = 0.0026, figure 2f) with the fastest median RTs for HighFP that were significantly faster than those of LowFP in both versions (version1: *p* = 0.012; version2: *p* = 0.035). Additional accuracy analyses for old and for new images can be found in the supplementary material along with individual data at https://osf.io/57u9w [40].

In summary, in both experiments 1 and 2, we found that the criterion for FP images was lower than for all other categories and especially lower than that of their control images (NoFP) and, interestingly, that it was modulated by the level of the face illusion (figure 2e).

### 3.3. Experiment 3

In experiments 1 and 2, we found that the decision criterion was significantly lower for FP images relative to their matched control images and that this effect was modulated by the magnitude of the induced face illusion. However, it was unclear whether this reflected a shift in decision criterion (response bias) or false recognition memory for illusory faces. Here we reduced the potential criterion bias (using a 2AFC paradigm) to examine whether our results reflected false recognition memory for illusory faces. False recognition memory for illusory faces was tested in the critical target-absent trials of this experiment (60 such trials out of 140 overall trials). These critical trials presented two unseen images (an unseen FP image alongside another unseen image from another category). It is important to note that responses in these critical trials could reflect either false memory or guessing behaviour (since these were not images that were seen before). We assumed that if false recognition for illusory faces exists, then participants would systematically favour the illusory-face image when forced to choose between that image and another image they had not previously seen. However, chance-level behaviour in the critical condition for all image categories would alternatively suggest that the results of experiments 1 and 2 are likely to be driven by criterion shift for illusory faces. We used illusory faces with high faceness ratings (all greater than or equal to 7, 7.78 ± 0.06 (M ± s.e.m.)), NoFP control images (not inducing an illusory face percept; all with faceness ratings = 0), face and outdoor images (Methods and figure 3a,b).

In the first cohort we ran, we found that memory accuracy for the valid trials for all categories was above chance (figure 3c; Faces: *t*(14) = 7.46, *p* = 1.5 × 10⁻⁶; FP: *t*(14) = 6.34, *p* = 9.2 × 10⁻⁶; NoFP: *t*(14) = 7.24, *p* = 2.2 × 10⁻⁶; Outdoors: *t*(14) = 6.19, *p* = 1.2 × 10⁻⁵; one-sample, one-tailed *t*-test). We also found an effect of category on accuracy (*F*(3,42) = 6.65, *p* = 9 × 10⁻^4^, *ŋ*^2^*_G_* = 0.134) that reflected higher memory accuracy for faces relative to outdoor images (*p* = 0.007). Importantly, in the critical trials, the proportion of FP choices was above chance only for the FP versus NoFP (*p* = 0.015; one-sample one-tailed *t*-test, figure 3d) but not for faces (*p* = 0.15) or for outdoors (*p* = 0.39). There was no effect of category on RTs in the valid trials ($\chi^{2}$(3) = 6.92, *p* = 0.074; figure 3e) and no differences between the RTs for the different categories in the critical trials ($\chi^{2}$(2) = 2.53, *p* = 0.28; figure 3f).

To test whether the effect of falsely remembering FP images more than NoFP images (in the critical trials) was genuine, we ran the second cohort. Here too, memory accuracy for the valid trials was above chance for all categories (Faces: *t*(15) = 5.08, *p* = 6.7 × 10⁻⁵; FP: *t*(15) = 5.74, *p* = 1.9 × 10⁻⁵; NoFP: *t*(15) = 5.69, *p* = 2.1 × 10⁻⁵; Outdoors: *t*(15) = 4.99, *p* = 8.0 × 10⁻⁵; one-sample, one-tailed *t*-test; figure 3c) and there was no effect of category on memory accuracy (*F*(3,45) = 2.16, *p* = 0.106, *ŋ*^2^*_G_* = 0.035). Importantly, in the critical trials, the proportion of FP choices was above chance for all categories (FP versus NoFP: *p* = 0.0001, FP versus faces: *p* = 0.015, FP versus outdoors: *p* = 0.0025; figure 3d). There was a significant effect of category on RTs for the valid trials ($\chi^{2}$(3) = 9.22, *p* = 0.026; figure 3e), though no individual post hoc comparison survived Bonferroni correction. The largest difference was between slower RTs for outdoors relative to FP (*p* = 0.063). In the critical trials, no differences between the RTs for the different categories were found ($\chi^{2}$(2) = 0.5, *p* = 0.78; figure 3f).

The effect of falsely remembering FP more than NoFP was replicated in the second cohort and also generalized to the other categories (FP falsely remembered more than faces or outdoor images). However, since both of these cohorts were small (16 participants each), we decided to run a third cohort of similar size to further substantiate (or refute) this finding.

The third cohort further replicated the main critical finding. Memory accuracy for the valid trials was above chance for all categories (Faces: *t*(16) = 5.77, *p* = 1.4 × 10⁻⁵; FP: *t*(16) = 9.25, *p* = 4.0 × 10⁻⁸; NoFP: *t*(16) = 6.27, *p* = 5.6 × 10⁻⁶; Outdoors: *t*(16) = 4.67, *p* = 1.3 × 10⁻⁴; one-sample, one-tailed *t*-test; figure 3c). There was an effect of category on memory accuracy (*F*(3,48) = 3.72, *p* = 0.017, *ŋ*^2^*_G_* = 0.073) that reflected higher memory accuracy for FP relative to outdoor images (*p* = 0.029). Importantly, in the critical trials, the proportion of FP choices was above chance for all categories (FP versus NoFP: *p* = 6.1 × 10⁻⁵, FP versus faces: *p* = 2.3 × 10⁻⁴, FP versus outdoors: *p* = 1.8 × 10⁻⁴; figure 3d). There was no effect of category on RTs in the valid trials ($\chi^{2}$(3) = 6.95, *p* = 0.073; figure 3e) and no differences between the RTs for the different categories in the critical trials ($\chi^{2}$(2) = 2.94, *p* = 0.23; figure 3f).

In summary, in experiment 3 we found, across three replications, that people tended to falsely report seeing FP images more than images of other categories when none of these images were viewed before. In addition, as each participant viewed a random set of images from each of the categories, this result is not likely to be influenced by specific images.

### 3.4. Experiment 4

Since we were also interested in obtaining additional measures of recognition memory strength for the valid and for the critical trials (overall and per category), in experiment 4 (‘cohort 4’) we relied on the same paradigm as in experiment 3 and in addition collected confidence ratings for each trial. Following the analysis we carried out in experiment 3, here we found that memory accuracy for the valid trials was above chance for all categories (Faces: *t*(19) = 9.55, *p* = 5.5 × 10⁻⁹; FP: *t*(19) = 7.86, *p* = 1.1 × 10⁻⁷; NoFP: *t*(19) = 10.5, *p* = 1.2 × 10⁻⁹; Outdoors: *t*(19) = 6.64, *p* = 1.2 × 10⁻⁶; one-sample, one-tailed *t*-test; figure 3c). We also found an effect of category on accuracy (*F*(3,57) = 3.8, *p* = 0.015, *ŋ*^2^*_G_* = 0.067) that reflected higher memory accuracy for NoFP relative to outdoor images (*p* = 0.012). Importantly, in the critical trials, the proportion of FP choices was above chance only when presented alongside NoFP (*p* = 0.027; one-sample one-tailed *t*-test, figure 3d) but not when presented alongside face (*p* = 0.21) or outdoor (*p* = 0.06) images. There was a significant effect of category on RTs in the valid trials ($\chi^{2}$(3) = 9.36, *p* = 0.025) that reflected slower RTs for outdoors relative to FP (*p* = 0.026; figure 3e), and no differences between the RTs for the different categories were found in the critical trials ($\chi^{2}$(2) = 0.7, *p* = 0.705; figure 3f).

Since the purpose of this experiment was to determine whether increased false alarms for unseen illusory faces reflect genuine memory-related processes (and not merely a criterion shift), we examined the self-reported confidence levels. We first anticipated that confidence for valid trials (that included an already-seen image alongside an unseen image and thus potentially elicited a real memory trace) would have higher confidence levels than that of the critical trials (presenting two unseen images) that could not elicit a real memory trace. In line with our assumption, valid trials' confidence levels (3.51 ± 0.13 (M ± s.e.m.)) was significantly higher than those of critical trials (2.58 ± 0.18; *t*(19) = 6.39, *p* = 1.99 × 10^–6^, paired one-tailed *t*-test; figure 3g). We also assumed and found that confidence for correct valid trials would be higher than for incorrect valid trials (correct valid trials confidence levels (3.73 ± 0.11) was significantly higher than those of incorrect valid trials (2.52 ± 0.19): *t*(19) = 6.9, *p* = 6.9 × 10^–7^, paired one-tailed *t*-test, figure 3h).

Falsely reporting FP images, as seen earlier, may also reflect false familiarity effects. We estimated whether confidence ratings were similar for genuine memory traces (correct valid FP trials) and for false memory reports (choosing FP in critical trials). Here we found that confidence ratings for correctly remembered FP images in the valid trials were rather high (4.16 ± 0.11 (M ± s.e.m.)), whereas for falsely remembered FP images in the critical trials confidence ratings were significantly lower (2.67 ± 0.18), *t*(19) = –8.86, *p* = 3.55 × 10^–8^, paired two-tailed *t*-test, figure 3i), reflecting low-to-moderate confidence that probably reflected familiarity effects [45,46].

We also wanted to examine whether people are more confident that they saw an FP image than images from other categories. To examine this for false memories, we compared the confidence levels for false memories for FP images with that of other categories. Indeed, we found that the confidence levels in the critical condition were higher for FP images (2.67 ± 0.18 (M ± s.e.m.)) than for other categories (2.45 ± 0.18): *t*(19) = 2.7, *p* = 0.014 (paired two-tailed *t*-test, figure 3j). To examine that for real memories, we tested whether this was also the case for correctly remembered images in the valid trials. There was an effect of category on confidence ratings (*F*(3,57) = 12.36, *p* = 2.46 × 10^–6^, *ŋ*^2^*_G_* = 0.211; one-way repeated ANOVA, figure 3k) that reflected higher confidence ratings for FP images relative to both NoFP (*p* = 0.004) and outdoors (*p* = 2.2 × 10⁻⁵), and this was marginally significant also relative to faces (*p* = 0.077). These results indicate that there is enhanced confidence when reporting remembering illusory faces relative to other categories, both for false memories (figure 3j) and for real memories (figure 3k).

We further wanted to test the possibility that the FP choice in the critical 2AFC trials was owing to the novelty of the other image (from the other category) rather than to the memory (or familiarity) of the FP image itself. To that end, we reanalysed RTs from experiment 1 that included the same categories as in this experiment and importantly, presented each image separately. We assume that RTs indirectly reflect confidence levels [44], and therefore if in the critical trials the novelty signal was stronger than the false FP memory/familiarity signal, then RTs of the correctly rejected (novel) images from experiment 1 would be faster than RTs of the false alarms for FP images. However, we found that there was no significant difference between the RTs (version1*:* FP false alarms versus Faces correct rejections: *w* = 254, *p* = 0.65; FP false alarms versus NoFP correct rejections: *w* = 233, *p* = 0.57; FP false alarms versus Outdoors correct rejections: w = 368, *p* = 0.12; version2*:* FP false alarms versus Faces correct rejections: *w* = 560, *p* = 0.84; FP false alarms versus NoFP correct rejections: *w* = 463, *p* = 0.4; FP false alarms versus Outdoors correct rejections: w = 536, *p* = 0.84; two-sided Wilcoxon signed-ranks test, figure 3l). These results hint that the novelty of the other categories was not stronger than the false memory/familiarity of new FP images. This suggests that the above chance choices of reporting seeing FP images in the critical trials probably reflected genuine false memory or familiarity.

### 3.5. Human versus ResMem memorability

It has recently been suggested that machine intelligence can mimic human abilities and especially human vision. Therefore, here we examined whether ResMem, an artificial intelligence neural network trained to predict image memorability from input images [36,47], yields memorability estimates aligned with human-based memorability scores. We relied on the human data obtained from experiment 3 since this was the only experiment where an image could have both hits and false alarm rates. We first estimated image memorability for experiment 3 images based on our participants' performance, and this can be seen by image category in figure 4a. In figure 4b are the ResMem image memorability estimations for experiment 3 images by category. As can be seen, in both human and machine vision, the only evident memorability difference was the lower memorability of outdoor images relative to other categories (one-way repeated ANOVAs, human: *F*(3,246) = 13.54, *p* = 3.31 × 10^–8^, *ŋ*^2^*_G_* = 0.142; all post hoc *p*’s (outdoor versus other category) < 0.003; ResMem: *F*(3,469) = 171.21, *p* = 5.91 × 10^–75^, *ŋ*^2^*_G_* = 0.523, all post hoc *p*’s (outdoor versus other category) < 10^–45^). We also directly compared, for each image, the human and ResMem memorability scores and found that they were significantly correlated (*r* = 0.38, *t*(248) = 6.49, *p* = 4.53 × 10^–10^; figure 4c).

**Figure 4.**
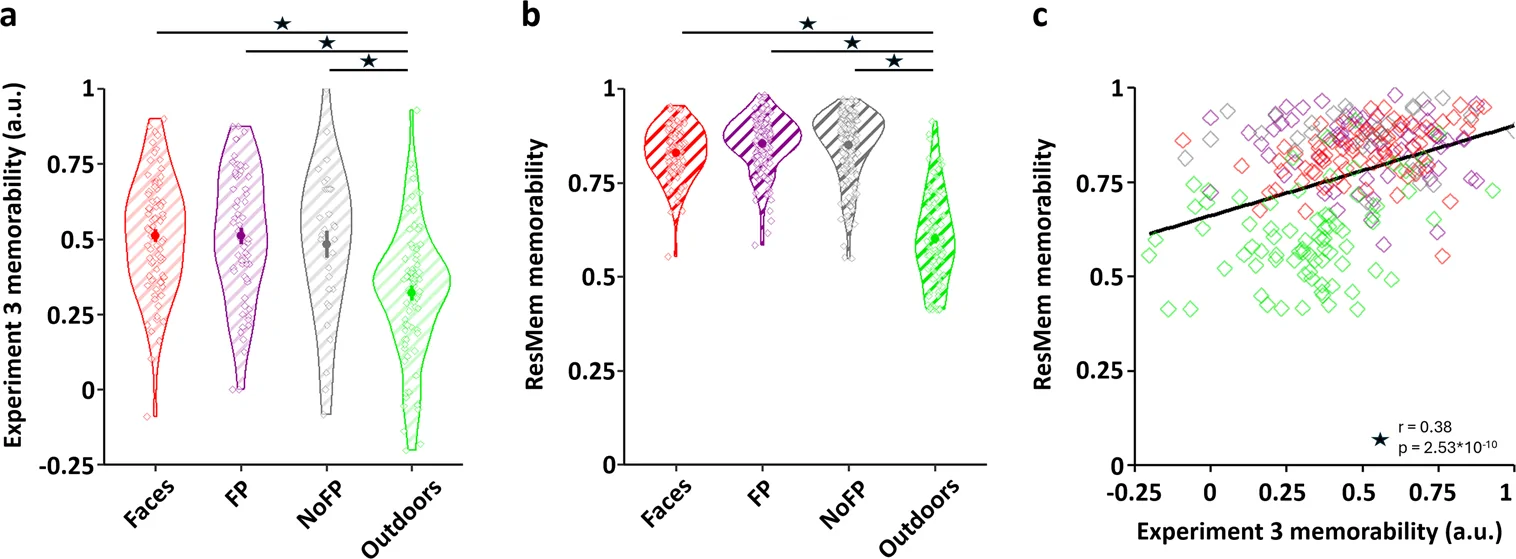
Human and ResMem memorability show similar category-related sensitivities. (a) Human memorability as estimated from experiment 3 data (y-axis; arbitrary units, a.u.) by visual category (x-axis) shows that memorability for outdoors was lower than that of the other categories. No differences were found between the memorability scores of face, illusory face and control images. (b) ResMem memorability scores of experiment 3 images also showed that memorability for outdoor images was lower than that of other categories, which had comparable memorability scores. (c) Human-based and ResMem memorability scores were significantly correlated.

## 4. Discussion

In this study, we took advantage of a widespread subjective illusory percept that is consistent across people and tested whether non-face images that elicit an illusory percept of a face, FP, are remembered more than content-matched control images or more than human face images. This followed our assumption that illusory face images are surprising, interesting, draw more attention and may beassociated with longer observation times. We also assumed that the recruitment of both face- and non-face-related brain mechanisms to process illusory faces may contribute to memory. In a set of experiments employing naturalistic encoding mimicking daily vision, we found that this was not the case and illusory faces were not remembered more than their control-matched images or more than human faces. However, unexpectedly, we found that people were more likely to report that they had seen an illusory face image before (i.e. a lower criterion or response bias) compared with images from other categories. In addition, this effect scaled with illusion strength such that people's response bias decreased for stronger examples of illusory faces compared with weaker examples. In follow-up 2AFC experiments that overcame potential criterion biases, we found people tended to falsely report seeing illusory faces more than other categories, even when neither image had been seen before. Confidence ratings indicated that people were more confident when reporting they remembered illusory faces relative to other categories and this was true for real memories and for false memories. Our results indicate that illusory faces bias recognition memory performance at the retrieval stage, and this may be associated with higher confidence levels or potentially a false sense of familiarity that illusory faces induce relative to images of other categories.

Several theoretical considerations led us to predict that memory for illusory faces would be enhanced relative to other image categories, although our findings do not provide conclusive evidence that this prediction was supported. *First*, Illusory faces may draw more attention and top-down processing as they may be more interesting to look at, and this may also be associated with longer observation times scanning them. *Second*, illusory face images are rather unusual in our visual experience and are novel, unfamiliar and surprising. Thus, while a face percept is elicited, the knowledge that this is not a face persists, and this ambiguity may lead to rival competing higher-order representations that may hinder prediction error reduction (e.g. [48,49]) and may prolong the processing of that image. *Third*, memory is assumed to be stronger for images and events that create more associations, and here the illusory face component itself creates an additional association, thereby potentially contributing to the memory strength of that image. *Fourth*, the neural mechanisms recruited to process illusory images also include face-related networks [17,32], and these may lead to higher overall activations and network connectivities associated with representing these stimuli. *Fifth*, many of the illusory faces are perceived as having an emotional expression [18], and this may also elicit responses in the network supporting emotional face perception and also in creating an emotional affective memory trace for that non-face image (note, however, that some of the human face images used in our experiment also conveyed an emotional expression). It may also be the case that illusory faces have a broader diversity of emotions and/or convey more intense expressions than typical face stimuli do [50]. *Sixth*, illusory face stimuli are different exemplars drawn from very different object categories (e.g. tree, cake and appliances), and this is in contrast to all the real face stimuli that are from the same visual category. It has been shown that higher conceptual distinctiveness contributes to higher memory. Thus, since illusory face images are much more conceptually distinct than faces, it is reasonable to assume they may be remembered better [51]. Taken together, these considerations indicate that there were sound theoretical reasons to expect enhanced memory for illusory faces. Instead, we found that people tend to report seeing illusory faces more than images from other categories regardless of whether they saw these illusory face images or not. Our results suggest that these factors either do not substantially improve memory or are counterbalanced by other processes under the present conditions. Future work will be needed to determine whether these factors are weaker than anticipated, counterbalanced by other processes or become apparent only under different experimental conditions.

Our findings reveal that the decision bias was influenced by illusory faces while memory sensitivity was not. This dissociation was evident by *d*' not being consistently modulated by visual category (faces, illusory faces and matched controls) or by the level of the faceness illusion, while response criterion shifted significantly toward a more liberal threshold as the level of the faceness illusion increased. Our finding is not likely to be only attributed to a simple response bias, since in the subsequent 2AFC critical condition, when both alternatives were foils, the illusory face images were selected above chance over foil images from other categories. Following the dual-process accounts of recognition memory [46,52,53], we assumed that the false recognition performance for illusory faces we found may reflect genuine false memories where people are strongly convinced they have seen these stimuli before, or otherwise false familiarity reflecting a sense that they have experienced these stimuli, yet without high conviction. To test this, additional control analyses, including confidence ratings, indicated that illusory faces probably induced higher false familiarity than that for other categories. There are several factors that may lead to higher false familiarity for illusory faces. First, participants may have responded to a diffuse, category-level familiarity signal that did not discriminate between studied and unstudied illusory faces. These results are consistent with the Fuzzy Trace Theory, which holds that memory is encoded along a continuum from verbatim item-specific traces to gist-level representations, with gist predominating when stimuli share strong categorical or perceptual overlap as may be the case with the illusory faces [54,55]. This may imply that many members of the face illusion class may activate the class ensemble representation and generate a non-specific familiarity response [56]. Second, the extent of illusory face images participants were exposed to in this study was probably higher relative to that of everyday exposure, and this may have affected decision criterion in a similar manner to the effects of base-rate influences (e.g. [57]). In addition, the level of perceptual vividness or fluency with which illusory stimuli are processed may have generated an inflated sense of familiarity at the decision stage, lowering the evidence threshold for recognition responses without enhancing the discriminability [58–60]. While we cannot determine precisely the factors contributing to the effect of illusory faces on false familiarity and decision criterion, our findings indicate that illusory faces influence recognition memory at the level of the retrieval stage rather than at the memory encoding stage.

The finding that ResMem memorability scores were correlated with human memorability scores may seem surprising given that our stimuli included an illusory category that ResMem was apparently not trained to capture. There are several explanations that may have contributed to this association. One possibility is that the overall correlation is driven by a gap in memorability scores between outdoor images and those of the other categories [4,61] that was evident in both humans and ResMem. Another possibility is that the illusory percept did not influence the memorability scores of both ResMem and humans, but for different reasons. For ResMem, memorability was not influenced by the illusory percept since it was not trained to be sensitive to such perceptual effects. For humans, memorability may actually reflect a lack of memory sensitivity to the illusory percept (relative to faces or content-matched control images). Lastly, the fact that our face and outdoor images were taken from the LaMem dataset [4], which ResMem was trained on, may have also contributed to the correlation despite the fact that our experiment included only a very limited set of images relative to the numerous images on LaMem. It is important to emphasize that our result may reflect the limited number of images that were included in our analysis, and sensitivity to illusory faces may arise in human and/or ResMem memorability with a larger illusory face set as has been shown earlier [62]. While our results do not explain the mechanisms underlying machine or human memorability, they further provide support for potential machine-to-human memorability associations.

There are several issues that may limit the generalization of our findings. The fact that our experiment included a high rate of illusory faces may have influenced our findings. In addition, we cannot determine whether our findings may generalize to other illusory stimuli or are limited to illusory faces. There may have also been conceptual interactions or interferences between the illusory faces and their content-matched controls images [51]. While our results were replicated across a set of experiments, bigger cohorts with higher statistical power may allow for more reliable memorability analyses that here were based, for some of the images, on as low as six participants' performance per image. We did not control here for the emotional content or arousal aspects of the images (faces and illusory faces alike) and therefore cannot determine whether these may have affected our results and to what extent.

The dissociation we found between memory sensitivity and criterion bias argues against a simple recognition memory process where stronger memory is associated with ‘stronger signal’ and weaker memory with ‘weaker signal’. Our findings suggest that illusory faces influence the recognition system at the retrieval stage independently of the actual memory trace (*d*'). Furthermore, they indicate that this influence is modulated by the strength of the perceptual illusion. Our results may have potential implications for how we think about the relationship between perceptual experience and memory judgements. They suggest that the subjective sense of seeing something beyond what is physically present may leave a trace that influences subsequent memory decisions, even when the underlying memory representation itself is not clearly stronger or weaker. This raises broader questions about the degree to which subjective perceptual experience, rather than objective stimulus properties alone, shapes the way we evaluate and report our memories.

## Data Availability

The data that support the findings of this study are available in the OSF repository [40].
